# Identification of genome-wide copy number variations among diverse pig breeds by array CGH

**DOI:** 10.1186/1471-2164-13-725

**Published:** 2012-12-24

**Authors:** Yan Li, Shuqi Mei, Xuying Zhang, Xianwen Peng, Gang Liu, Hu Tao, Huayu Wu, Siwen Jiang, Yuanzhu Xiong, Fenge Li

**Affiliations:** 1 Key Laboratory of Pig Genetics and Breeding of Ministry of Agriculture & Key Laboratory of Agricultural Animal Genetics, Breeding and Reproduction of Ministry of Education, Huazhong Agricultural University, Wuhan, 430070, PR China; 2 Hubei Key Laboratory of Animal Embryo Engineering and Molecular Breeding, Hubei Academy of Agriculture Science, Wuhan, 430070, PR China

## Abstract

**Background:**

Recent studies have shown that copy number variation (CNV) in mammalian genomes contributes to phenotypic diversity, including health and disease status. In domestic pigs, CNV has been catalogued by several reports, but the extent of CNV and the phenotypic effects are far from clear. The goal of this study was to identify CNV regions (CNVRs) in pigs based on array comparative genome hybridization (aCGH).

**Results:**

Here a custom-made tiling oligo-nucleotide array was used with a median probe spacing of 2506 bp for screening 12 pigs including 3 Chinese native pigs (one Chinese Erhualian, one Tongcheng and one Yangxin pig), 5 European pigs (one Large White, one Pietrain, one White Duroc and two Landrace pigs), 2 synthetic pigs (Chinese new line DIV pigs) and 2 crossbred pigs (Landrace × DIV pigs) with a Duroc pig as the reference. Two hundred and fifty-nine CNVRs across chromosomes 1–18 and X were identified, with an average size of 65.07 kb and a median size of 98.74 kb, covering 16.85 Mb or 0.74% of the whole genome. Concerning copy number status, 93 (35.91%) CNVRs were called as gains, 140 (54.05%) were called as losses and the remaining 26 (10.04%) were called as both gains and losses. Of all detected CNVRs, 171 (66.02%) and 34 (13.13%) CNVRs directly overlapped with *Sus scrofa* duplicated sequences and pig QTLs, respectively. The CNVRs encompassed 372 full length Ensembl transcripts. Two CNVRs identified by aCGH were validated using real-time quantitative PCR (qPCR).

**Conclusions:**

Using 720 K array CGH (aCGH) we described a map of porcine CNVs which facilitated the identification of structural variations for important phenotypes and the assessment of the genetic diversity of pigs.

## Background

Genetic and archaeological findings suggest that pig domestication began about 9000–10000 years before present (YBP) at multiple sites across Eurasia, followed by their subsequent spread at a worldwide scale [1]. Historically, Europe and China are two major areas of pig breeding [2]. Over the past centuries, pigs have shown marked differences between these two areas, even if many European pig breeds carry far Eastern haplotypes at high frequencies because of an ancient introgression with Chinese swine [1]. The Chinese pigs differ significantly from European pig breeds such as the Large White for many traits including fatness and ear traits [3-5]. Genetic variation within the gene pool which produce the above different phenotypes are selected for or against by evolution. Microsatellites, single nucleotide polymorphisms (SNPs) were the main measures of genetic variations in pigs, producing a USMARC pig SNP map (http://www.marc.usda.gov/genome/swine/marker_list.html) and the PorcineSNP60 Genotyping BeadChip with 62163 SNP probes [6]. Recently, structural variations including insertions, duplications, deletions, inversions and translocations of DNA have been shown to contribute to the major phenotypic variations [7]. Copy number variation (CNV) is described as a segment of DNA >1 kb that is copy number variable when compared with a reference genome [8]. This variation may either be inherited or caused by de novo mutation [9-12]. It has become apparent that CNVs are genome-wide present in the human genome [8] and the genome of farm animals including cattle [13-16], avian [17-19], sheep [20], goat [21]. About a range from 5% to 16% of the human genome was covered by CNVs [22,23]. CNVs can lead to striking phenotypic consequences as a result of altering gene dosage, disrupting coding sequences, or perturbing long-range gene regulation by position effects [24-26]. These striking phenotypic consequences include some common complex diseases such as autism [11], schizophrenia [12], auto-immune Addison's disease [27].

Recently many efforts have been used to detect pig CNVs. By a custom-made tiling oligonucleotide array, 37 CNV regions (CNVRs) across chromosomes 4, 7, 14, and 17 were identified in 12 unrelated Duroc boars [28]. Comparative genome hybridization (CGH) array was also conducted for chromosomes 7 and 8 in 9 different pig populations including Duroc, Large White, Meishan, Pietrain, Hampshire and Wild Boar [29]. By analyzing data from the Porcine SNP60 BeadChip, 49 CNVRs were identified in 55 animals from an Iberian × Landrace cross (IBMAP) [30] and 382 CNVRs were identified from three purebred populations (Yorkshire, Landrace and Songliao Black) and one Duroc × Erhualian crossbred population [31]. Up until now, few studies have confirmed the genome-wide presence of CNVs in pigs using array CGH (aCGH) with high-density probes. Here we reported the use of high-resolution oligonucleotide aCGH to identify the CNV regions in 12 individual pigs from different pig populations. This analysis provided a high-resolution map of copy number variations in the pig genome with a median probe spacing of 2506 bp relative to the latest porcine genome assembly (Sscrofa9.2).

## Results and discussion

### The overview of CNVR library

**Figure 1:**
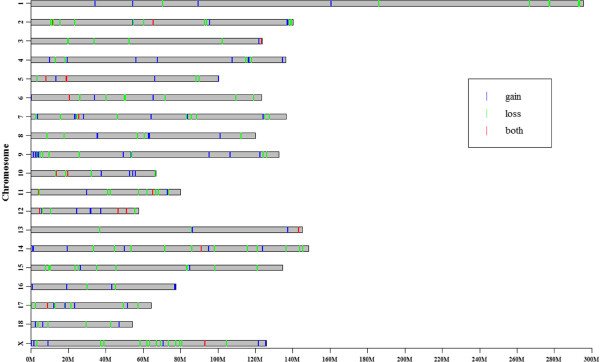
**Graphical representation of the CNVRs.** Blue lines represent gain predicted status, losses are indicated in green, and regions with both gains and losses status are represented in red. X axis values are chromosome position in Mb. Y axis values are chromosome names. Chromosome sizes are represented in proportion to the real size of the *Sus scrofa* karyotype obtained from the Ensembl database.

**Figure 2:**
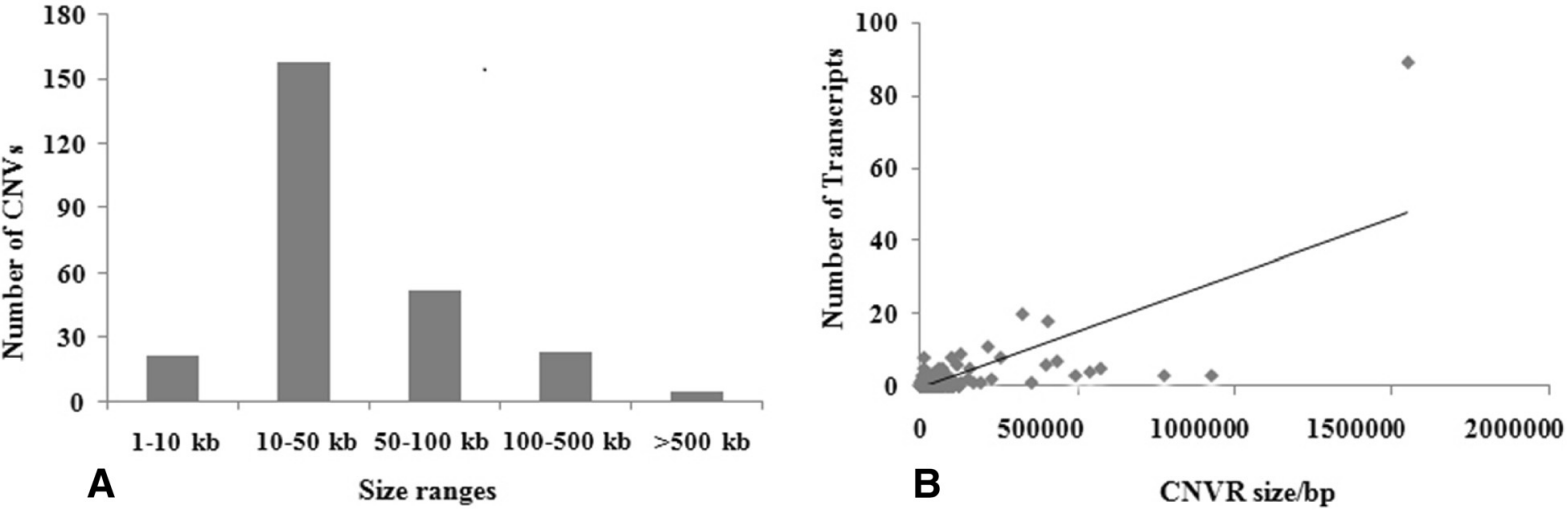
**CNVR characteristics.A:** Size range distribution of the CNVRs; **B:** Number of transcripts in CNVRs.

Array CGH (NCBI GEO accession no. GPL16165) was carried out using a custom-made array comprising 719,336 oligonucleotide probes covering the whole pig genome assembly with a median probe spacing of 2506 bp (Additional file 1). CNV was assessed by equating the log2 ratio of signal intensity between the reference (Duroc) and test samples. As we did not perform a self-to-self experiment, a stringent criterion with the mean |log2 ratio| > 0.5 was used to reduce the false positive rate of CNV calling according to the studies of Wang et al. [19] and Fadista et al. [28]. Therefore, the segments with at least 5 consecutive probes and a mean |log2 ratio| of > 0.5 were merged [28,32]. A CNVR was then called if detected in two or more animals. Accordingly, we identified 259 CNVRs (Figure 1, Additional file 2). The CNVRs ranged in size from 2.30 kb to 1.55 Mb with a mean of 65.07 kb and a median of 98.74 kb, covering 16.85 Mb or 0.74% of the whole genome (Figure 2A, Additional file 2). The largest CNV region, CNVR_85 with 1.55 Mb in size on chromosome 7, showed copy gain in the White Duroc pig, the Pietrain pig, 2 Landrace × DIV pigs and loss in the Yangxin pig and the Large White pig.

Using the custom tiling oligonucleotide aCGH approach, Fadista et al. [28] addressed 37 CNVRs on the *Sus scrofa* chromosomes (SSCs) 4, 7, 14, and 17 of the preliminary assembly of pig genome among 12 Duroc boars. Ramayo-Caldas et al. [30] detected 49 CNVRs using the Porcine SNP60 BeadChip data of 55 animals from an Iberian × Landrace cross. Wang et al. [31] detected 382 CNVRs based on the Porcine SNP60 genotyping data of 474 pigs. Two of the 37 CNVRs (5.41%) detected by Fadista et al. [28], 8 of the 49 CNVRs (16.32%) detected by Ramayo-Caldas et al. [30], 24 of the 382 CNVRs (6.28%) detected by Wang et al. [31] were identical or overlapped with the detected CNVRs in this study (Additional file 2). Totally 39 of the presently detected 259 CNVRs (15.06%) were identical or overlapped with those previously reported pig CNVRs (Additional file 2). The main potential reasons for this less well-overlapping result could be the different genetic backgrounds of pig samples, different platforms and various calling algorithms between the present study and other studies.

Compared with PorcineSNP60 Genotyping BeadChip, the detection power of 720 K aCGH was enhanced by dense marker density, uniform distribution of probes along each chromosome [6,30]. Hence, some small CNVRs can be detected by aCGH technique, as the minimum CNV lengths were 2.30 kb in our present study, and 2.08 kb in the study of Fadista et al. [28], whereas the minimum CNV length detected by SNP chip were 5.03 kb and 44.65 kb, respectively [30,31].

### CNVRs chromosome distribution and status

CNVRs were distributed throughout the genome in a non-random manner (Additional file 2), which was coherent with the previous studies on heterogeneous distribution of CNVs in primate genomes [9,14]. Chromosomes 2, 7, 10–12 and 17 had the dense CNVs covering more than 1.00% of genomic sequences (Table 1). A conserved synteny between *Homo sapiens* chromosome 17 (HSA17) and SSC12 had been proposed (https://www-lgc.toulouse.inra.fr/pig/compare/SSC.htm). Proportional to its length, HSA17 was especially rich in primate-specific breakpoint regions which would appear to be highly enriched for both segmental duplications (SDs) and CNVs [33,34].

**Table 1:** Chromosome distribution of CNVRs in pigs

**Chr.**	**No. CNVRs**	**CNVR Size (bp)**	**Chr. Size (bp)**	**Percentage (%)**
1	12	1158170	295534705	0.392
2	19	1524080	140138492	1.088
3	9	757763	123604780	0.613
4	13	416439	136259946	0.306
5	13	542338	100521970	0.539
6	11	363611	123310171	0.295
7	19	2572973	136414062	1.886
8	12	1185818	119990671	0.988
9	18	927039	132473591	0.699
10	13	1673511	66741929	2.507
11	12	1220486	79819395	1.529
12	16	885260	57436344	1.541
13	7	198599	145240301	0.137
14	18	665744	148515138	0.448
15	15	407233	134546103	0.303
16	8	325153	77440658	0.419
17	14	869490	64400339	1.350
18	9	360816	54314914	0.664
X	22	798761	125876292	0.634
Total	259	16853284	2262579801	0.745

Concerning copy number status, 93 (35.91%) CNVRs were called as gains, 140 (54.05%) were called as losses and the remaining 26 (10.04%) were called as both gains and losses. Previously, it has been suggested that deletions are under stronger purifying selection than duplications [35]. If so, deletions should be both less frequent and shorter than duplications [14]. However, when we compared the length of gains with losses in the CNVRs, loss regions had slightly larger sizes than gain regions with the average length of 57.39 kb and 45.86 kb respectively (T-test not statistically significant at p value > 0.05). The possible reason was that the aCGH approach might favor the identification of deletions [14,15,21,28]. As the samples were collected from 9 different populations, the considerable number of CNVRs status displaying in ‘both gains and losses’ might be due to the different genetic origins.

### Putative population-specific CNVRs and cluster analysis

Some putative population-specific CNVRs were detected. For example, 6 CNVRs including CNVR_132 were purebred Landrace-specific, and CNVR_145 were purebred DIV-specific. CNVR_100 including *KIT* gene contained amplifications specifically in 8 pigs with dominant white color and a Pietrain pig with black spots, and CNVR_251 contained gains in pigs without dominant white color such as Yangxin, Erhualian, Tongcheng and Pietrain pigs. However, due to the limited samples used in the present study, the putative population-specific CNVRs need future study. And we also found 3 de novo CNVRs, of which CNVR_IDs 36, 149 were present in 2 Landrace × DIV crossbred pigs but not in their parents, while CNVR_259 were absent in 2 Landrace × DIV crossbred pigs but present in their parents.

**Figure 3:**
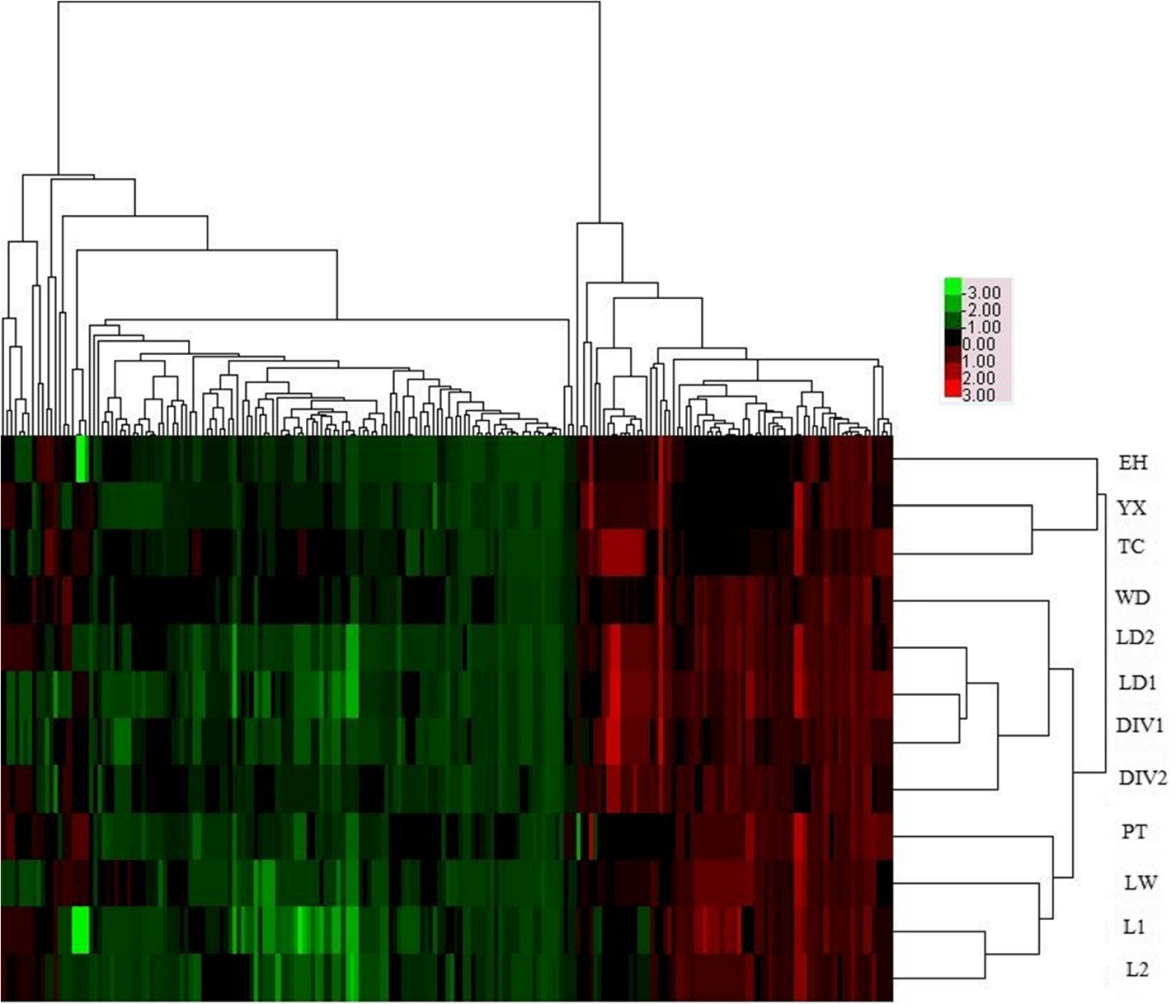
The dendrogram of 12 pigs generated by average linkage clustering algorithm of Cluster 3.0 software.

Using the cluster tool, average linkage hierarchical clustering based on the CNV profiles of 12 tested pigs was performed. Figure 3 showed the dendrogram of 12 pigs generated by average linkage clustering algorithm of Cluster 3.0 software. Basically, the Chinese native pigs (Erhualian, Yangxin, Tongcheng) clustered together, while the other 9 pigs with European haplotypes belonged to another big cluster. Therefore, CNVs could be used to investigate pig genetic diversity and evolution.

### Duplicated sequences colocalize with CNVRs in the pig genome

Although the exact interpretation of mechanisms responsible for generating CNVs is still unclear, previous studies have noted a four- to twenty-fold enrichment of CNVs near SDs in the other mammalian genomes [22,32,36]. Duplicated sequences are typical segments of DNA which range in size from one to hundreds of kb, share a high level of sequence identity (≥ 90%) and occur at more than one site within the genome [28]. Under the same filter criterion, about 66.02% (171/259) of CNV regions directly overlapping with *Sus scrofa* duplicated sequences were identified through blasting the CNVR sequence against the Ensembl pig genomic sequences. As our present BLAST results did not retain a CNVR overlapping with a duplicated sequence by less than 1000 bp, so the overlaps of CNVs and their targeted duplicated sequences were under reporting. There were 13.5–25.0% CNVRs mapped to duplicated sequences in the previous reports [28,37]. The difference may be related to differences in samples. CNVRs overlapping duplicated sequences were significantly different in average size (87.12 kb versus 22.23 kb, t-test p < 0.01) with the CNVRs that did not overlap duplicated sequences, consistent with previous CNV studies reporting a stronger association between duplicated sequences and long CNVRs [9,11].

### Gene contents of pig CNV regions

When CNV signals in two or more animals overlapped on a chromosome, they were considered to be high confidence CNVs [19]. Presently, the high confidence CNVRs contained transcripts from 0 to 89. The largest region (CNVR_5) detected in all tested pigs showed an 87.21 kb gain without overlapping any gene or duplicated sequence (Additional file 2). Same as the previous report in chicken [19], our results showed the small CNVs resided in none coding sequences, while larger CNV regions spanned more genes (Figure 2B, Additional file 2). The 259 CNVRs encompassed 372 unique transcripts which corrsonded 154 mouse orthologous genes annotated in Ensembl (Additional file 3). In order to determine the likely biological effects of the 154 mouse orthologous genes, functional annotation analysis was performed with the DAVID tool [38]. Gene Ontology (GO) analysis revealed that CNVR genes belonged to these classes of genes that participated in sensory perception of smell, sensory perception of smell or chemical stimulus, sensory perception, cognition, G-protein coupled receptor protein signaling pathway, olfactory receptor activity and other basic metabolic processes (Table 2). KEGG pathway analyses indicated that 50 genes involved in olfactory transduction (p < 0.05) were over-represented in the porcine CNVRs, as previously identified in cattle [15,31,37]. These CNV genes also included ATP-binding cassette, sub-family C (CFTR/MRP), tyrosine-protein kinase Kit (*KIT*) and cytochrome P450 (*CYTP450*) as described previously [30,37]. A certain degree of conservation of CNVs across mammals has been observed, which suggests that selective pressure may drive acquisition or retention of specific gene dosage alterations.

**Table 2:** Enriched GO terms and KEGG pathway associated with the CNV regions (Modified Fisher Exact P-value ≤ 0.05)

**Category**	**Term**	**Count**	**Involved genes/ total genes (%)**	**P value**	**Validation**
GO_BP	GO:0007608 ~ sensory perception of smell	52	35.14	1.5E-29	Pig [31]
GO_BP	GO:0007606 ~ sensory perception of chemical stimulus	52	35.14	3.3E-28	Pig [31]
GO_BP	GO:0007600 ~ sensory perception	53	35.81	6.8E-26	Pig [31]
GO_BP	GO:0050890 ~ cognition	53	35.81	8.9E-25	Pig [31]
GO_BP	GO:0007186 ~ G-protein coupled receptor protein signaling pathway	56	37.84	1.2E-22	Pig [31]
GO_BP	GO:0050877 ~ neurological system process	53	35.81	3.4E-22	Pig [31]
GO_BP	GO:0007166 ~ cell surface receptor linked signal transduction	60	40.54	6.1E-20	Pig [31]
GO_MF	GO:0004984 ~ olfactory receptor activity	55	37.16	1.6E-28	Pig [31] Cattle [14]
GO_MF	GO:0047961 ~ glycine N-acyltransferase activity	2	1.35	0.025	
GO_CC	GO:0016021 ~ integral to membrane	77	52.03	4.5E-6	Pig [31]
GO_CC	GO:0031224 ~ intrinsic to membrane	78	52.70	9.3E-6	Pig [31]
KEGG_PATHWAY	mmu04740:Olfactory transduction	50	33.78	8.8E-19	Pig [31]

To test whether genes unaffected by CNVs exhibited a different selective constraint than the ones affected, we compared the dN/dS ratios for orthologous genes of pigs with those of mouse and human species (Table 3, Additional file 3). Compared with mouse, all pig CNVR genes had dN/dS ratios significantly higher than monomorphic genes by Wilcoxon rank-sum test, which was the same as the previous results [14]. It might indicate a relaxation of purifying selection due to the redundancy fragments generated during the formation process of the variable number of genes [39-42]. However, compared with mouse, the pig CNVR genes with the status of gains had dN/dS ratios lower than monomorphic genes, indicating these genes subjected to stringent purifying selection compared with non-polymorphic genes.

**Table 3:** Evolutionary rates of pig monomorphic and CNVR genes compared with human and mouse

		**CNVR(gain)**	**CNVR(loss)**	**CNVR(both)**	**No CNVR**
Human	dN/dS	0.20	0.32	0.27	0.21
	P value	2.41E-03	3.82E-12	1.08E-06	-
Mouse	dN/dS	0.87	0.33	0.22	0.19
	P value	1.72E-07	2.20E-16	2.14E-10	-

dN: nonsynonymous rate; dS : synonymous rate; p-value compares CNVR genes with monomorphic genes by Wilcoxon rank-sum test.

### Pig CNVRs overlapped with QTL regions

We queried the animal QTL database that held publicly available QTL data on livestock species. Retrieving all the porcine QTLs (http://www.animalgenome.org/cgi-bin/QTLdb/SS/download?file=gbpSS_10.2) within 2 Mb of our CNVRs resulted that 34 CNVRs overlapped with QTLs for several important traits including average daily gain (ADG) (Additional file 4). However, as the pig QTLs are not fully defined, the contribution of these QTL-overlapping CNVRs to complex traits needs further study.

### Validation of CNVRs by real-time quantitative (qPCR)

qPCR was performed to validate 2 CNVRs (CNVR_IDs 100 and 215) detected by the aCGH experiment. Thirteen DNA samples including the reference used in aCGH were used for qPCR analysis. CNVR_100 and CNVR_215 were validated (Additional file 5) with the p threshold values 0.05 as the previous reports [43].

**Figure 4:**
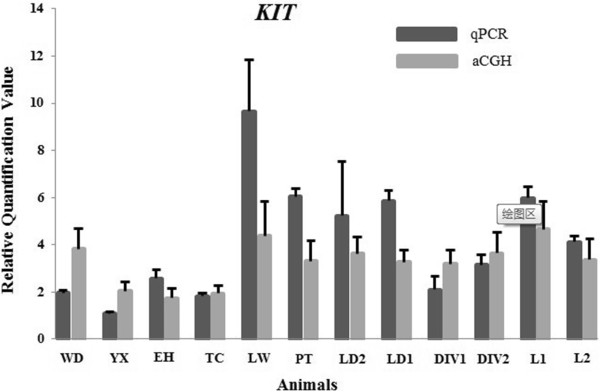
**Validation of CNVR _100 (*KIT *gene) detected from the CGH array data using real-time quantitative PCR analysis.** The x-axis represents the animals and the y-axis shows the relative quantification value (2^-ΔΔCt^ values for qPCR; 2*(2^Sample signal) values for array CGH).

CNVR_100 contained Mast/stem cell growth factor receptor gene, also known as *KIT* gene (ENSSSCT00000009679). In pigs, the dominant white color was associated with a splice mutation leading to the skipping of exon 17 of *KIT* gene [44] and a duplication of a 450 kb fragment encompassing the *KIT* gene [45]. The results of the aCGH array and qPCR analyses revealed that the copy number varied greatly among the different breeds (Figure 4). Coinciding with the previous study [45], 8 pigs with white hair color (one White Duroc pig, one Large White pig, two Landrace × DIV pigs, two Landrace pigs and two DIV pigs) and the Pietrain pig had *KIT* duplication, but 3 Chinese native pigs without pure white color did not have. In addition to the important role in proliferation, survival and migration of melanocytes [45], the *KIT* gene also had effects on follicle and oocyte development [46,47]. Therefore, it was worthy to further investigate the selection impact of white hair color on pig reproduction traits.

## Conclusions

In summary, we described a map of porcine CNVs between breeds by a high-resolution array CGH, which was confirmed to be a very valid method to detect porcine genome-wide CNVs. With a stringent CNV calling criterion, 259 highly reliable CNV regions were reported here among diverse pig breeds. Future studies are required to assess the function of CNVs on pig important phenotypes. Our results facilitated the identification of structural variations for important phenotypes and the assessment of the genetic diversity in pigs.

## Methods

### Sample preparation

All animal procedures were performed according to protocols approved by the Biological Studies Animal Care and Use Committee of Hubei Province, PR China. Twelve pigs including one White Duroc pig (♀), one Chinese Yangxin pig (♂), one Chinese Erhualian pig (♀), one Chinese Tongcheng pig (♀), one Large White pig (♀), one Pietrain pig (♂), two Landrace pigs (♂), two DIV pigs (♀) and two Landrace × DIV pigs (♀, ♂) were selected to function as test animals. Chinese Erhualian pigs were a strain of Chinese Taihu pig breed. Synthetic Line DIV was a result of cross of Landrace, Large White, Tongcheng or Taihu pigs. An unrelated female Duroc pig was selected as the common reference. The genomic DNA of 13 pig samples was extracted and purified from semen, whole blood or ear notch.

### Oligonucleotide aCGH

A 3 × 720 K whole genome tiling aCGH (NCBI GEO accession no. GPL16165) was designed (NimbleGen Systems, http://www.nimblegen.com) from the Sscrofa9.2 release (http://www.sanger.ac.uk/Projects/S_scrofa/), which was the new release at the time of the experiment. The probe design fundamentals were described in the NimbleGen technical note (http://www.nimblegen.com/products/lit/probe_design_2008_06_04.pdf). The probes with length of 50–60 bp were integrated into an array design using ArrayScribeTM, which resulted in a design with a median probe spacing of 2506 bp. Test DNA and reference DNA samples were independently labeled with either Cy3 or Cy5 dyes. Labeled DNA was co-hybridized to the custom-made NimbleGen CGH array (3 × 720 K). The array format included 3 arrays on single slides containing 719,336 probes. The arrays were scanned using a 5 μm scanner, and NimbleScan software (Roche NimbleGen) was used to retrieve fluorescent intensity raw data from the scanned images of the oligonucleotide tiling arrays. For each spot on the array, log2 ratios of the Cy3-labeled test sample versus Cy5-labeled reference sample were computed. Before normalization and segmentation analysis, spatial correction was applied. Specifically, locally weighted polynomial regression (LOESS) was used to adjust signal intensities based on X, Y feature position [48]. Normalization was then performed using the q-spline method followed by segmentation using the CNV calling algorithm segMNT included in NimbleScan software [11]. CNVRs were called as the segments with at least 5 consecutive probes, a mean |log2 ratio| of >0.50 and detected in two or more animals [28]. Since the CNV calling pipeline requires at least 5 consecutive probes, our theoretical resolution for CNV detection is 10299 bp (median spacing × 4 + median oligo length × 5). As females had two copies of X-linked genes and males only had one copy, male–female aCGH resulted in an excess of female signals for X-linked genes that can be used to calibrate the threshold values and detection methods [49]. aCGH data have been submitted to the GenBank gene expression omnibus database under the accession number GSE41488. The dendrogram were generated by average linkage clustering algorithm of Cluster 3.0 software [50].

### Enrichment analysis

In order to check if the CNVRs overlapped any duplicated sequence, BLAST was used to query the CNVRs sequences against the *Sus scrofa* genome sequence (Sscrofa9.2). Sequences were retained as duplicated sequences if they had ≥ 1 kb and ≥ 90% identity and occurred at more than one site within the genome.

Gene contents in the identified CNVRs were retrieved from the Sscrofa9.2 assembly using the BioMart (http://www.biomart.org/) [51]. Gene content of pig CNV regions was assessed using Ensembl transcripts. The DAVID functional annotation tool (http://david.abcc.ncifcrf.gov/) was used to perform GO classification and KEGG pathway annotation of CNV mRNAs. Functional annotation terms from the ontologies of "biological processes", "molecular function" and "cellular component" were recorded. Since only a limited number of genes in the pig genome have been annotated, we converted the pig Ensembl transcripts IDs to orthologous mouse and human Ensembl gene IDs by BioMart, then carried out the GO and pathway analyses, as described previously [31].

All the porcine QTLs data were downloaded from pig QTL database (http://www.animalgenome.org/cgi-bin/QTLdb/SS/download?file=gbpSS_10.2) [52]. The CNVRs were considered to be overlapping pig QTLs if they were within 2 Mb of pig QTLs [14].

### Validation of CNVRs by qPCR

Determination of CNVRs by qPCR was performed using the Roche LightCycler® 480 Detection System and obtained the crossing thresholds (Ct) value following the guidelines of the manufacturer. The primers were designed using the Primer Premier 5 software and were available in the Additional file 6. As previously reported [28], the copy number of each CNVR was normalized against the Col10 region, a control region in the genome that did not vary in copy number between the pigs. Triplicate wells of reactions (15 μL) contained 7.5 μL SYBR Green Real-time PCR Master Mix, 1 μL of 10–20 ng/μL gDNA, 0.3 μL 5 μM of each primer and 0.1 μL ROX. The cycling conditions consisted of 1 cycle at 95°C for 10 min, followed by 40 cycles at 94°C for 20 sec, 60°C for 20 sec, and 72°C for 20 sec, with fluorescence acquisition at 74°C in single mode. The specific PCR products were confirmed by the results of melting curve analysis and agarose gel electrophoresis. Analysis of resultant crossing thresholds (Ct) was performed using the -ΔΔCt method [53].

## Abbreviations

CNV: Copy number variation; CNVR: CNV region; PCR: Polymerase chain reaction; CGH: Comparative genome hybridization; aCGH: Array CGH; qPCR: Real-time quantitative PCR; RQ: Relative quantification value; QTL: Quantitative trait locus; KIT: Tyrosine-protein kinase Kit; CYTP450: Cytochrome P450 gene family; SNP: Single nuclotide polymorphism; HSA: *Homo sapiens* chromosome; SSC: *Sus scrofa* chromosome; GO: Gene ontology; DAVID: The database for annotation, visualization and integrated discovery; KEGG: kyoto encyclopedia of genes and genomes; LOESS: locally weighted polynomial regression; Ct: crossing thresholds; SD: Segmental duplication.

## Competing interests

The authors have declared that no financial competing interests exist.

## Authors' contributions

YL, SM, FL carried out most of bioinformatics analysis and lab works. XZ, XP, HW, GL, HT participated in the animal samples collection and statistical analysis. FL, SJ, YX participated in the experiment design and coordination. FL conceived the study and drafted the manuscript. All authors read and approved the final manuscript.

## Supplementary Material

Additional file 1 Probe summary of the 720 K custom-made CGH array designed by Roche NimbleGen. Click here for file

Additional file 2 **Description of the CNVRs detected by a whole-genome CGH array.** The genomic coordinates were expressed in bp and were relative to the *Sus scrofa* genome sequence assembly (Sscrofa9.2). BLAST was used to query the CNVRs sequences against the *Sus scrofa* genome sequence (Sscrofa9.2). Sequences were retained as duplicated sequences if they had ≥ 1 kb and ≥ 90% identity and occur at more than one site within the genome. WD: White Duroc (♀); YX: Yangxin (♂); EH: Erhualian (♀); TC: Tongcheng (♀); LW: Large White (♀); PT: Pietrain (♂); LD1: Landrace × DIV pig 1 (♂); LD2: Landrace × DIV pig 2 (♀); DIV1: Chinese new pig line DIV 1 (♀); DIV2: Chinese new pig line DIV 2 (♀); L1: Landrace 1 (♂); L2: Landrace 2 (♂). Click here for file

Additional file 3 Gene contents of CNVRs. Click here for file

Additional file 4 **QTLs overlapped with the CNVRs.** All the porcine QTLs within 2 Mb (http://www.animalgenome.org/cgi-bin/QTLdb/SS/download?file=gbpSS_10.2) of our CNVRs were counted. Click here for file

Additional file 5 The validation of the aCGH results using qPCR method. Click here for file

Additional file 6 The primers of qPCR to validate the CNVRs detected by aCGH. Click here for file
